# Asymmetries in Infants’ Vowel Perception: Changes in Vowel Discrimination in German Learning 6- and 9-Month-Old Infants

**DOI:** 10.1177/00238309241228237

**Published:** 2024-02-19

**Authors:** Antonia Götz, Anna Krasotkina, Gudrun Schwarzer, Barbara Höhle

**Affiliations:** Universität Potsdam, Germany; MARCS Institute for Brain, Behaviour, and Development, Western Sydney University, Australia; Universität Potsdam, Germany; Justus-Liebig Universität Giessen, Germany; Justus-Liebig Universität Giessen, Germany; Universität Potsdam, Germany

**Keywords:** Vowel perception, perceptual reorganization, asymmetry, infants’ speech perception

## Abstract

Infants’ speech perception is characterized by substantial changes during the first year of life that attune the processing mechanisms to the specific properties of the ambient language. This paper focuses on these developmental changes in vowel perception. More specifically, the emergence and potential cause of perceptual asymmetries in vowel perception are investigated by an experimental study on German 6- and 9-month-olds’ discrimination of a vowel contrast that is not phonemic in German. Results show discrimination without any asymmetry in the 6-month-olds but an asymmetrical pattern with better performance when the vowel changes from the less focal to the more focal vowel than vice versa by the 9-month-olds. The results concerning the asymmetries are compatible with the Natural Referent Framework as well as with the Native Language Magnet model. Our results foster two main conclusions. First, bi-directional testing must be mandatory when testing vowel perception. Second, when testing non-native vowel perception, the relation of the stimuli to the native language vowel system has to be considered very carefully as this system impacts the perception of non-native vowels.

## 1 Introduction

Infants’ development of speech perception involves an increased impact of properties of the native language(s) during the first of life. This shaping from potentially innate acoustic biases toward the attunement to specific properties of the native language(s) is called perceptual reorganization. One prominent feature of this developmental change is that infants’ initial ability to discriminate speech sounds is maintained or even enhanced for native sounds but weakened for non-native speech sounds (Aslin & Pisoni, 1980). Perceptual reorganization has been shown to affect the perception of consonants (Rivera-Gaxiola et al., 2005; Werker & Tees, 1984), lexical tones (Götz et al., 2018; Liu & Kager, 2014; Mattock & Burnham, 2006; Mattock et al., 2008; Yeung et al., 2013), lexical stress (Höhle et al., 2009; Skoruppa et al., 2009), and vowels (e.g. Polka & Bohn, 1996, 2011; Polka & Werker, 1994; Tsuji & Cristia, 2014).

This study focuses on the development of vowel perception, especially on the question of what asymmetries in vowel perception (i.e., if, for example, a change from Sound X to Y is better noticed by a listener than a change from Y to X) reveal about the interplay of acoustic biases and language experience in early development. To this end, German-learning 6- and 9-month-olds were tested on their discrimination of a vowel contrast that is non-phonemic in German. Our main question was whether a perceptual change would happen across these ages and whether asymmetries in the perception of these vowels occur and whether such asymmetries change across ages.

A meta-analysis of 22 different studies on infant vowel discrimination that were published until 2012 revealed that vowel perception starts to be affected by language experience from the age of 6 months on: the effect sizes of measures for infants’ discrimination of native vowel contrasts increase significantly between 6 and 10 months, while the effect sizes of measures for non-native vowel contrasts decrease (Tsuji & Cristia, 2014). This suggests the typical pattern of perceptual reorganization with enhancement of the perception of sound contrasts that are phonemic in the respective language but weakening of the perception of sound contrasts that are not. This finding is further corroborated by neurophysiological studies with German-learning infants showing a development toward adult-like native vowel processing from 6 to 10 months (Schaadt et al., 2023). However, a decline of perceptual sensitivity for non-native vowel contrasts was not found in all studies (de Klerk et al., 2019; Mazuka et al., 2014; Polka & Bohn, 1996, 2011) which may relate to the fact that potential asymmetries in vowel discrimination have not been paid attention to in all studies. Sound discrimination in infants is typically tested by experimental setups in which one element of a sound pair serves as background stimulus and the infants’ response to a change from the background stimulus to the other element of the pair is measured. In habituation or familiarization paradigms, the background stimulus is repeatedly presented during an initial exposure phase while the contrasting sound is presented in the test phase. In oddball paradigms, the contrasting element is spread into a sequence of background sounds. Importantly, some—but not all—previous studies have tested changes in both directions by counterbalancing which sound was selected as background or contrasting stimulus and these studies revealed a modulation of infants’ response by the direction of change (for a review see Polka & Bohn, 2011).

To account for the observed asymmetries, Polka and Bohn (2003, 2011) proposed the Natural Reference Vowel (NRV) framework. According to the most recent versions of this framework, a phonetic property of vowels—focalization—is relevant for predicting asymmetries. Focalization refers to the convergence of two adjacent formants: if two adjacent formants (either F1 and F2 or F2 and F3) are close together, spectral energy is concentrated in a narrow field. The closer the two formants are, the more focal is the vowel. According to NRV, a focal vowel acts as an anchor point and perceptual salience increases toward the more focal vowel. Discrimination is easier from the less to the more focal vowel than vice versa. The most focal vowels are /a/, /i/, /u/, and /y/ (Polka and Bohn, 2011; Schwartz et al., 2005, 1997). The asymmetries in vowel perception predicted by the NRV framework are considered to be universal and thereby initially language-independent as they are grounded in the phonetic properties of the vowels and the phonetic-acoustic biases that guide infants’ vowel processing. However, the perceptual asymmetries may be modulated by native language experience (Masapollo, Polka, & Ménard, 2017; Polka & Bohn, 2011). As Polka and Bohn (2011, page 474/475) state,

. . . vowel perception biases (and their related directional asymmetries) will be maintained or enhanced for non-native vowel contrasts but will be reduced or absent for native vowel contrasts in many phonetic processing tasks. . . . This shift begins in infancy and will continue until the native phonology is consolidated.

Following this proposal, asymmetries based on phonetic-acoustic biases are expected to manifest themselves at an earlier age and are independent from language development. Furthermore, developmental shifts are expected to influence the presence of perceptual asymmetries in distinct ways for native and non-native vowel contrasts. Specifically, asymmetries in native vowels may diminish, whereas those in non-native vowels could maintain or enhance throughout development.

However, research on native vowel perception throughout development revealed mixed findings with respect to asymmetries. Polka and Bohn (1996) tested 6- to 8- and 10- to 12-month-old English- and German-learning infants on their discrimination between the German vowels /u/ and /y/ and on their discrimination of the English /æ/-/ԑ/ contrast. Neither age nor native language modulated the results: in both age groups, discrimination was better when testing the change from /y/ to /u/ and from /æ/ to /ԑ/ than in the other direction. However, their study groups were rather small such that statistical power may have been too low to reveal differences. In contrast, two studies with larger groups of infants have found developmental changes in asymmetric effects in native vowel perception. Polka and Bohn (2011) report data from Danish learning 6- to 12-month-olds’ discrimination of two pairs of Danish vowels, /e/-/ԑ/ and /e/-/ø/. They found better discrimination in the direction from /ԑ/ to /e/ and from /e/ to /ø/ than in the other direction in the younger infants. However, this asymmetry was not evident anymore in the older infants. Pons et al. (2012) found that Catalan- and Spanish-learning 4- and 6-month-old infants discriminated the—for both languages native—vowel /e/-/i/ contrast better in the direction from /e/ to /i/ than vice versa. However, language-specific asymmetrical effects were found in the group of 12-month-olds: Catalan-learning infants showed asymmetrical effects in the same direction as the 4- and 6-month-olds (from /e/ to /i/) but the Spanish-learning 12-month-old infants showed increased discrimination in the opposite direction (from /i/ to /e/). The authors concluded that after the perceptual reorganization process, language-specific effects such as frequency of vowel occurrence (which is different for the two vowels in Catalan and Spanish) restructure asymmetrical vowel perception. In summary, the evidence regarding asymmetrical perception of native vowel contrasts suggests a possible reduction in asymmetries as individuals develop, acknowledging that other factors, such as the frequency of vowels within the native phonological system, may influence asymmetrical perception.

Similar to the perception of native vowels, language-specific factors are proposed to have an influence on the perception of non-native vowels. With respect to potential changes of asymmetrical perception of non-native vowel discrimination throughout development, the first study reporting asymmetrical effects was conducted by Polka and Werker (1994). They tested English infants’ ability to discriminate the German front–back vowel contrast (/u/-/y/) between the ages of 4 and 10 months. Their results showed better discrimination when tested from /y/ to /u/ than vice versa with this asymmetry being stronger in 6- to 8-month-olds than in 10- to 12-month-olds. As the /u/ was more similar to an English /u/ than the /y/, Polka and Werker (1994) explained this asymmetry as being driven by the exposure to the English language, thus reflecting language-specific perception abilities. As mentioned above, Polka and Bohn (1996) did not find effects of language background or age in their study with German and English learning infants: the infants for which the respective vowel contrast was non-native showed the same direction of asymmetry as the infants for which the contrast was phonemic in their native language. So far, only Polka and Bohn (2011) provide evidence for different developmental trajectories in the occurrence of perceptual asymmetries for native compared with non-native vowel contrasts. In addition to the Danish vowels, they included the English (/ɜ/–/e/) contrast which is non-native for the Danish-learning infants. The study revealed a robust asymmetry in the discrimination of the non-native vowels that did not change across the ages (while the asymmetry in the perception of the native Danish vowels had disappeared across the ages).

Further evidence suggests that asymmetries in the discrimination of non-native vowel contrasts may vary across the specific vowel pairs tested. Mazuka and colleagues (2014) investigated the perception of German vowel contrasts in Japanese infants and found asymmetries that diminished or emerged depending on the contrast. In their study, 4.5-month-olds but not 10-month-olds tended to show a perceptual asymmetry for the non-native /u:/-/y:/ contrast, with better discrimination from /u:/ to /y:/ than vice versa. The opposite developmental pattern was found for the /i:/-/e:/ contrast. Here, the 10-month-olds showed better discrimination in the direction of /i:/ to /e:/ than vice versa, whereas no asymmetry was found in 4.5-month-old infants. These findings with Japanese infants’ non-native vowel perception are not compatible with the NRV: first, the observed asymmetries are in the wrong direction, and second, they show opposite developmental patterns with a disappearance of the asymmetry for the /u:/-/y:/ and an appearance for the /i:/-/e:/ contrast. Noteworthy, Mazuka and colleagues (2014) point to a crucial factor that has not systematically been considered in studies of infants’ vowel perception: the relation of the vowels tested to the native vowel system that may lead to the perceptual assimilation of the perceived vowel to a native vowel category.

According to the Perceptual Assimilation Model (PAM, Best, 1994, 1995), the processing of non-native sounds entails the mapping of the sounds to the native language phonetic system. Mainly studied for consonant perception before, Tyler et al. (2014) showed that these mapping processes are evident also in vowel processing: adults’ non-native vowel discrimination was harder when the two vowels were perceived as equally good exemplars of a single native vowel category than when they were mapped to two different native categories. Interestingly, asymmetric effects were also modulated by assimilation: they only occurred when the contrasting sounds were rated as exemplars of a single native vowel category but not when they were rated as exemplars of different native vowel categories.

Hence, if the non-native vowels are assimilated to a single native speech sound category, properties of this native category should affect processing. Here comes a second account into play that considers asymmetries in the perception of vowels within a native category: the Native Language Magnet model (NLM, NLM-e, Kuhl, 1991; Kuhl et al., 2006, 2008; Kuhl & Iverson, 1995). This framework assumes that asymmetries in within-category vowel perception are based on the language-specific internal structure of a vowel category. According to this approach, native language sound categories have prototypical exemplars that act as perceptual magnets by attracting other members of the category. Therefore, better performance in detecting a change from a less prototypical to the prototypical vowel than in the other direction is expected. Indeed, adults and infants from 6 months of age showed perceptual asymmetries consistent with this approach (Grieser & Kuhl, 1989; Kuhl, 1991). Kuhl et al. (1992) further showed that this asymmetry is stronger for native vowels than for non-native vowels in 6-month-olds. This supports the assumption that asymmetric perception is a product of infants’ acquisition of the native language-specific internal structure of a vowel category.

### 1.1 The present study

The goal of this study was to further investigate the developmental trajectory of vowel perception and to shed further light on asymmetrical perception and its development for which previous research does not show a consistent picture yet. We tested 6- and 9-month-old German infants’ discrimination of the vowel /ɪ/ and the vowel /ɨ/. This contrast is non-phonemic in German. However, due to the phonetic similarity between the two vowels, we expected the vowel /ɨ/ to be assimilated to the German /ɪ/ category. A study with German-speaking adults confirmed this expectation and showed in addition that the /ɪ/ was rated as a much better exemplar of this category than the vowel /ɨ/. For the infant experiment, our expectations vary based on the specific theoretical framework. Following the NRV framework, we predict better performance from /ɨ/ to /ɪ/ in both the 6-month-olds and 9-month-olds as the /ɪ/ is the more focal vowel compared with /ɨ/ (Sanders & Padgett, 2008). Furthermore, the NRV predicts that this asymmetry should already be evident in the younger age group since, for example, Polka and Bohn (2011) have found language-independent asymmetries at this age. However, taking PAM into account, we assume that 9-month-olds assimilate the vowels to their native /ɪ/ category (as suggested by the many findings indicating a decrease in discriminating non-native contrasts at that age). Given that the two vowels are perceived as members of the same vowel category that differ in their typicality for this category (with /ɪ/ being the more prototypical exemplar than /ɨ/), the NLM (Kuhl et al., 2008) would also predict better performance from /ɨ/ to /ɪ/ than vice versa. Thus, the NLM predicts the same direction of asymmetry with better performance from /ɨ/ to /ɪ/ as the NRV, but according to the NLM, the asymmetry is expected to get stronger across ages since it reflects language-specific properties of the vowel space.

## 2 Methods

### 2.1 Participants

In total, 80 German-learning infants participated in this study, 40 six-month-olds (20 female, *M* = 181 days, range = 162–205 days) and 40 nine-month-olds (20 female, *M*_age_ = 272 days, range = 261–288 days). Infants were recruited from a database of parents who had expressed interest in participating with their child in research. An additional 21 infants were tested but were excluded from further analyses for the following reasons: crying (*n* = 6: three 6-month-olds, and three 9-month-olds), fussiness (*n* = 1, 9-month-old), not reaching the habituation criterion (*n* = 11: six 6-month-olds, and five 9-month-olds), and technical error (*n* = 3: all 6-month-olds).

According to the parental report, participating infants did not suffer from repeated or acute ear infections, and there were no indications of atypical development. All infants were born full-term and had no hearing deficits. The study was carried out in accordance with the recommendations of the Ethics Committee of the University with written informed consent given by the parents.

### 2.2 Stimuli

**Figure 1.**
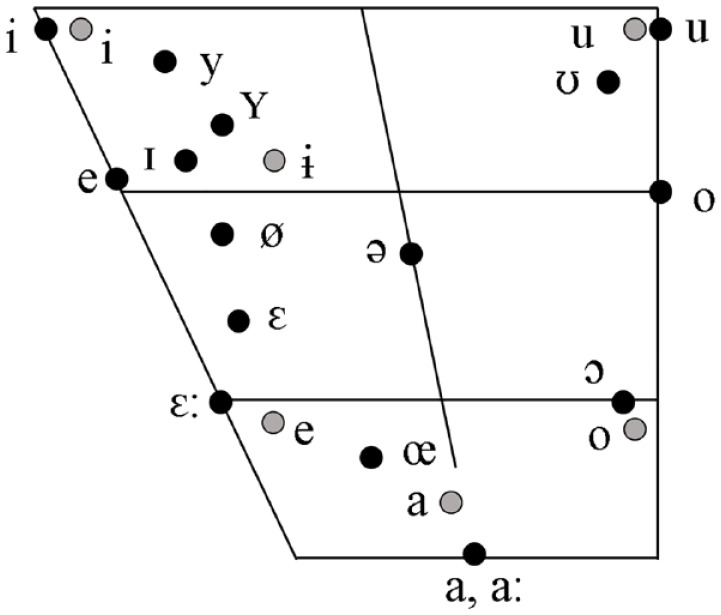
Polish and German vowel chart. *Note*. Vowel chart with the comparison of the Polish (gray) and the German vowel (black) system. Modified according to Kohler (1999) for German, and Jassem (2003) for Polish vowels.

A balanced bilingual speaker of Polish and German was recorded to create our stimuli. In a language questionnaire, the bilingual speaker indicated a balanced amount of written and oral communication using Polish and German in her daily life. In addition, we recorded two female native monolingual speakers of Polish and German. The bilingual speaker produced the stimuli in both languages, and the monolinguals in their respective native language. The recordings of the monolingual speakers were utilized to choose the appropriate vowels from the recordings of the bilingual speaker, ensuring that the produced vowels closely resembled native-like pronunciations. A comparison of German and Polish vowels is provided in Figure 1. Six tokens of the vowels (/ɪ/ and /ɨ/, respectively) were recorded in CVC syllables (/pVk/, and /tVk/) in isolation as well as embedded in either Polish or German sentences. The stimuli were presented to the speakers in written form. In Polish, the vowel /ɨ/ was orthographically presented as < y > (e.g., < pyk >, and < tyk >), whereas the German vowel was presented as < pick >, and < tick >, which marks orthographically the vowel /ɪ/.

The stimuli were recorded and digitalized at 44.1 kHz with Audacity in a soundproof booth. The crucial syllables were then spliced from the utterances and acoustic measures of the vowel formats (F1 and F2 frequencies) were obtained via PRAAT. The stimuli used in this study were selected from the productions of the bilingual speaker on the basis of the frequencies of the first two formants which should not differ more than 1 *SD* from the productions of the respective monolingual speaker. The acoustic measures of the two selected tokens from each language are displayed in Table 1.

**Table 1.** Acoustic Measurements of the Vowels.

Stimuli	Duration (ms)	Vowel duration (ms)	F0 (Hz)	F1 (Hz)	F2 (Hz)	F3 (Hz)	F4 (Hz)
/t ɨ k/ token1	363	68	233 (224–245)	461	2184	2952	3523
/t ɨ k/ token2	361	88	246 (245–251)	474	2195	2922	3523
/t ɪ k/ token1	357	75	219 (212–235)	433	2296	2844	3501
/t ɪ k/ token2	348	81	224 (222–229)	445	2295	2804	3647

*Note.* Acoustic measurements of the vowels in the four syllables were used as stimuli in the experiment.

The four tokens selected for the use in the experiment were then submitted to a rating task, in which 15 German-monolingual adults were asked to identify the selected vowels and rate their goodness as an instance of a German vowel. The stimuli were presented three times mixed with instances of three other German vowels (/u/, /y/, and /i/). In the identification task, participants were asked to decide whether they heard the native phonemic vowels /ɪ/, /ə/, /i/, /u/, or /y/. The corresponding vowels were orthographically displayed on the screen. Before the experiment started, the match between the orthographical representation of the vowels and the corresponding sound was demonstrated to the participant. For example, < i > was presenting the /ɪ/ vowel, and < ie > the /i/ vowel, which is analogue to the German orthography, < e > was presented to represent /ə/ and participants received the additional information that this is the same vowel as in the underlined vowels in *eine Biene* (a bee). Participants had to indicate their response by pressing a button on the keyboard. Listeners identified both vowels to an almost identical percentage as belonging to the German vowel category (88% of the /ɨ/ productions and 89% of the /ɪ/ productions). Directly after the identification task, the category goodness rating was conducted. The participants were asked to indicate how typical/untypical the vowels sounded for a German vowel on a scale ranging from 1 (*no fit*) to 7 (*very good fit*) by pressing the corresponding button on the keyboard. The results revealed that the German adults rated the Polish vowel significantly less typical than the German vowel (*M* = 4.2 vs. 5.6; β = −0.47, *SE* = 0.09, *t* = −4.93, *p* < .001). Thus, the control study confirms that the selected German and Polish vowel exemplars are identified as instances of the same vowel category but that the German vowel is a better representative of this category for German listeners.

For the use in the infant experiment, the two tokens of a syllable with the same vowel then were concatenated in a random order to a 40-s speech string that included 30 instances of the syllables with an interstimulus interval of 1 s.

### 2.3 Procedure

Infants were tested in a visual fixation paradigm. During the experiment, they sat on their caretakers’ lap facing a monitor at a distance of approximately 1.2 m. Infants’ looking behavior was monitored and recorded by a camera positioned above the screen. Listening time was online coded via a button-box by an experimenter who sat behind a partition. The speech stimuli were presented using Habit2 (Version 2.1.25; Oakes et al., 2015) with an intensity of 65 dB over loudspeakers positioned behind the screen. During the presentation of the speech strings, a black-and-white checkerboard was displayed on the screen. A silent bouncing ball appeared on the screen during the intertrial interval between the presentation of the speech strings. As soon as the infant fixated the screen, the experimenter pressed a key and thereby started the presentation of the speech string. Trials ended when infants either looked away for more than 2 s or when the end of the speech string was reached.

The experiment consisted of three phases: habituation, test, and posttest phase. During the habituation phase, the infants were exposed to one of the strings with the syllables containing one of the vowels until a habituation criterion was reached. The habituation criterion was fulfilled when infants’ mean listening time across three consecutive trials had decreased to 50% of the mean listening time of the first three habituation trials. The maximum number of trials within the habituation phase was 18 trials. Half of the infants were habituated with the /tVk/ syllables containing the vowel /ɨ/ and the other half with the syllable containing the vowel /ɪ/. The test phase started immediately after the infant had reached the habituation criterion or after the maximum number of habituation trials had been presented. The test phase contained four trials: two trials presented the two exemplars of the habituated vowel; the other two trials presented the exemplars of the non-habituated novel vowel. The trial order was counterbalanced across infants. If infants discriminate between the two vowels, longer listening times to the novel compared with the habituated vowel are expected. The posttest phase followed directly after the test phase. During the posttest phase, a novel stimulus with a consonantal change from /tVk/ to /pVk/ was presented to verify infants’ attention to the auditory stimuli. In total, the video recordings of 25% of the participants (randomly selected) were offline re-coded (frame by frame, 25 fps) with ELAN by a second coder. The inter-coder reliability was *r* = .98, *p* < .001.

## 3 Data analysis

Only data from infants who had reached the habituation criterion within the 18 habituation trials were included in the analysis. Listening times were logarithmically transformed to achieve a normal distribution (see Csibra et al., 2016). In both age groups, there were no statistically significant differences in listening times for the two vowels during the habituation phase, 6-month-olds: *t*(39) = 1.114, *p* = .272; 9-month-olds: *t*(39) = −1.263, *p* = .215. In addition, there was no significant evidence to suggest that the 6-month-olds had greater exposure to either the /ɨ/ vowel or the /ɪ/ vowel compared with the 9-month-olds, /ɨ/: *t*(39) = 1.177, *p* = .247; /ɪ/: *t*(39) = 0.912, *p* = .367. To test the effects of age and direction of change on the listening times during the test trials, we compared different models that were computed with the lmer function from the lme4 package (Bates et al., 2015). The log-transformed listening times of the test trials were used as the dependent variable, and we included fix factors (the first model included Condition, with the two levels of habituated vs. novel vowel; the second model Condition and Age [6 and 9 months]; and the third model included Age, Condition, and Habituation vowel [/ɨ/ vs. /ɪ/]). Subsequently, we compared the models with their fit to the data. Subject was included as random factor, adding random slopes resulted in singular fits indicating that model was overfitted. The best fitting model included the interaction of Condition (habituated vs. novel vowel), Age (6 and 9 months), and Habituation (/ɨ/ vs. /ɪ/), which had the lowest Akaike information criterion (AIC, Akaike, 1998; *AIC* = 611.87), and was significantly different in chi-square, χ^2^(6) = 14.09, *p* = .029, compared with the model including only condition (with an AIC = 613.96) or the model including the interaction of Condition and Age (with an of *AIC* = 614.62). The output of the model comparisons is provided in Table 2. The precise model was lmer<- log(LT) ~ Condition*Age*Habituation + (1|Subject).

**Table 2.** Results From the Model Comparison.

Model	*df*	AIC	BIC	logLik	Deviance	Chisq	Chi *df*	Pr (>Chisq)
~ Condition + (1—Subject)	4	613.96	628.98	−302.98	605.96			
~ Condition*Age + (1—Subject)	6	614.62	637.16	−301.31	602.62	3.33	2	0.188
~ Condition*Age*Habituation + (1—Subject)	10	611.87	649.42	−295.93	591.87	14.09	6	0.029^a^

a *p* < .05.

*Note*. The comparison is hierarchically organized. The first model was compared with the second model—which fit not better to the data. The better model (the first model) was then compared with the third. Results from chi-square test and AIC score revealed the best model fit for the model which includes the interaction of Condition, Age, and Habituation as fixed effect and subject as random effect. Adding random slopes to the model led to singular fits. AIC = Akaike information criterion; BIC = Bayesian information criterion.

## 4 Results

**Figure 2.**
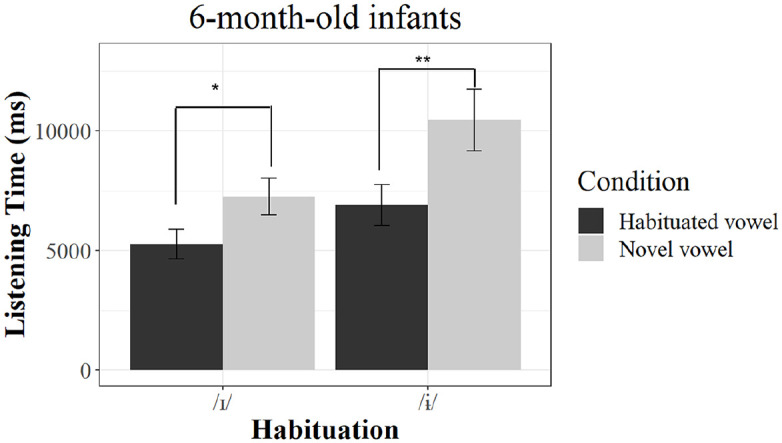
Results of listening times in 6-month-old infants. *Note*. Mean listening times to the novel and habituated vowels of the 6-month-olds per habituation group. Simple asterisk (*) represents *p* < .05, double asterisks (**) indicate *p* < .01. Error bars represent standard errors of the mean.

The results indicate a significant interaction of Condition, Age, and Habituation, χ²(2) = 6.242, *p* = .044, hence indicating developmental differences in listening times to the novel and habituated vowels depending on the vowel that was presented during the habituation phase. We followed up the interaction by pairwise comparison and the results revealed that the 6-month-old infants showed significantly longer listening times during the trials that presented the novel vowel compared with the trials that presented the habituated vowel independent from the vowel presented during habituation—habituation with /ɨ/: β = 0.3708, *SE* = 0.1241, *df* = 60, *t* = 2.988, *p* = .00407, effect size Cohen’s *d* ^1^ = 0.555, LT_novel_ = 10,464.25 ms (*SD* = 8,157.41), LT_habituated_ = 6,886.25 ms (*SD* = 5,464.13); habituation with /ɪ/: β = 0.2835, *SE* = 0.1248, *df* = 60, *t* = 2.272, *p* = .0267, Cohen’s *d* = 0.464, LT_novel_ = 7,255.05 ms (*SD* = 4,895.30), LT_habituated_ = 5,258.65 ms (*SD* = 3935.23), hence indicating no asymmetrical vowel perception, see Figure 2.

**Figure 3.**
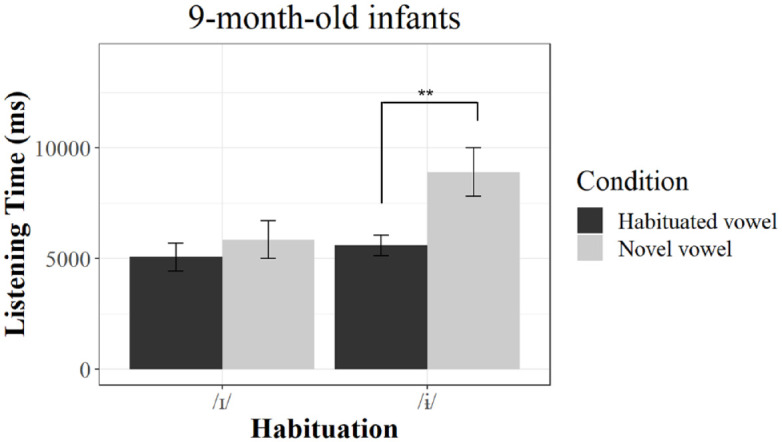
Results of listening times in 9-month-old infants. *Note*. Mean listening times to the novel and habituated vowels of the 9-month-olds per habituation group. Double asterisks (**) indicate *p* < .01. Error bars represent standard errors of the mean.

The 9-month-old infants showed significantly longer listening times to the novel vowel compared with the habituated vowel only when they were habituated with /ɨ/ (β = 0.3358, *SE* = 0.1129, *df* = 60, *t* = 2.973, *p* = .00424, Cohen’s *d* = 0.510, LT_novel_ = 8,901.30 ms [*SD* = 6,909.52], LT_habituated_ = 5,074.68 ms [*SD* = 3,916.47]), but not when they were habituated with /ɪ/ (β = 0.0711, *SE* = 0.1335, *df* = 60, *t* = 0.533, *p* = .596, Cohen’s *d* = 0.105, LT_novel_ = 5,850.90 ms [*SD* = 5,178.98], LT_habituated_ = 5,595.48 ms [*SD* = 2,896.08]), see Figure 3. Hence, asymmetrical vowel perception was evident, with a significant difference in listening times between the novel and habituated vowel only in the group that was habituated with the /ɨ/ vowel.

## 5 Discussion

This study aimed to investigate the developmental trajectory of vowel perception during the first year of life. Most importantly, we were interested in asymmetries in vowel discrimination and their potential developmental changes between the ages of 6 and 9 months. To this end, we tested potential developmental changes in vowel discrimination in German infants at 6 and 9 months of age and whether asymmetries in the discrimination of a within-category vowel contrast emerge across these ages.

Two main findings arise from our study. First, at 6 months, infants showed no asymmetrical performance: they discriminated the two vowels, and discrimination was found independently from the vowel used for habituation. Second, 9-month-olds showed an asymmetrical performance pattern with clear indications of discrimination only when habituated with /ɨ/ but not when habituated with the vowel /ɪ/.

Our findings emphasize that perceptual reorganization of vowels cannot simply be characterized by a decrease in discrimination ability for non-native sounds. Instead, it can be marked by a perceptual shift that becomes evident through the emergence of asymmetries in 9-month-old infants. Comparable to previous findings on infant speech perception, German-learning 6-month-olds showed a rather robust ability to discriminate between the two vowels. At 9 months, this ability was not lost but became more affected by the testing conditions as being dependent on the selection of habituation and test stimulus. Our results highlight the relevance of considering potential perceptual asymmetries when studying perceptual reorganization. Testing only one direction of change for the same vowel contrast would have yielded contrasting results. If we had solely examined a group habituated to the /ɨ/ vowel, the data would indicate a pattern of maintenance—the ability to discriminate tested would seemingly be unchanged across the ages tested. Conversely, if we had exclusively tested a group habituated to the /ɪ/ vowel, the data might have suggested a decline in discrimination ability. Hence, these findings may support our assumption that some of the inconsistent results on the perceptual reorganization of vowels may be caused by these kinds of asymmetries in perception. Further research thus needs carefully to consider these potential asymmetric effects in their experimental settings.

What do our results tell us about the underlying causes of asymmetries in vowel perception? Remember that the NRV (Polka & Bohn, 2011) considers the source of these asymmetries in the phonetic properties of the vowels with easier discrimination from a less focal to a more focal vowel than in the reverse direction (Masapollo et al., 2018; Masapollo, Polka, Molnar, & Ménard, 2017; Polka & Bohn, 2011). In development, the initial perceptual asymmetry can disappear for native vowel contrasts and be maintained for non-native contrasts. In contrast, the NLM (Kuhl et al., 2008) interprets asymmetrical perception as emerging with the establishment of language-specific vowel categories and their internal structure. According to this model, changes from a more prototypical exemplar to a less prototypical exemplar within a native vowel category are harder to detect than a change in the opposite direction. According to PAM (Tyler et al., 2014), the understanding of asymmetries in non-native vowel perception needs the knowledge of how the non-native vowels relate to the native vowel categories.

Coming first to the results of the German 6-month-olds who did not show evidence for asymmetries in their perception of the two vowels, based on previous results in the framework of the NRV, asymmetric perception with better discrimination from /ɨ/ to /ɪ/ was expected. However, it needs to be kept in mind that not all previous studies could confirm the predictions of the NRV for this age group by either not finding asymmetries at all (de Klerk et al., 2019; Mazuka et al., 2014) or finding asymmetries that go into the unexpected direction (Mazuka et al., 2014; Polka & Bohn, 2011). One factor contributing to these inconsistent results could be the case that the distinctness between the vowels selected for the specific study plays a role. In our study, the two vowels were rather close in the F1/F2 space but the acoustic distance was sufficient to discriminate the vowels. However, the two vowels may not sufficiently differ in focalization—the property that according to the NRV is relevant for the emergence of perceptual asymmetries. Since we observed asymmetrical perception in 9-month-olds for the same vowel contrast, we do not believe that the acoustic distinctiveness plays the major role in the results of the 6-month-olds but their still lower level of language experience. In any case, for a better understanding of the sources of potential asymmetries, distinctiveness between the vowels used as contrasts should be considered more closely in future research.

In light of the NLM, the results of the 6-month-olds can be interpreted in the following way. Remember that the original studies by Grieser and Kuhl (1989), Kuhl (1991), and Kuhl et al. (1992), showed that within-vowel category perception is already language-specific in 6-month-old infants. Hence, the lack of a perceptual asymmetry in the 6-month-olds tested in our study is not in line with NLM given that both vowels were categorized into the same vowel category by adult speakers of German but differed in their fit into the category (/ɪ/ was rated as a better exemplar for the German category than the /ɨ/ vowel). Nonetheless, expecting asymmetric perception on this vowel pair relies on the assumption that 6-month-old infants have already fully developed knowledge of the relevant vowel category. However, since there has been limited research on the development of the vowel system in German-learning infants, we lack information regarding the age at which language-specific effects can be anticipated. Furthermore, the meta-analysis conducted by Tsuji and Cristia (2014) suggests that infants begin to exhibit language-specific vowel processing after 6 months of age, but not prior to this milestone. Based on our findings, we would infer that at the age of 6 months, German infants do not yet exhibit responsiveness to the native internal structure of the vowel category to which adult speakers had assigned the two vowels.

This brings us to the results from the 9-month-olds and the importance of considering developmental trajectories. Unlike the 6-month-olds, the 9-month-olds showed an asymmetrical discrimination pattern for the two vowels. The direction of this asymmetry aligns with both theoretical accounts: the NRV hypothesis anticipates this specific asymmetry because the /ɨ/ vowel is less focal than the /ɪ/ vowel, while the NLM hypothesis aligns with this direction based on the ratings we obtained from the German adults. As previously mentioned, this latter conclusion draws on the assumption that German-learning infants have learned sufficiently about their vowel system to map the perceived sounds to this system and to have collected sufficient information about the internal structure of the vowel category used in our experiment. This assumption is also crucial for considering the results of the 9-month-olds in the light of the NRV as the NRV makes different predictions concerning the development of native and non-native perception. According to Polka and Bohn (2011), potential asymmetries will be maintained or enhanced for non-native and reduced or absent for native vowel contrasts. We do not assume that the 9-month-olds treat the vowels presented in our study as a native German contrast. Per definition, the contrast is non-native as it is contrastive in another language than German. Therefore, the NRV approach would predict maintenance or enhancement of asymmetrical perception. While the appearance of the asymmetry at 9-month-olds fits the NRV approach, the approach is less compatible with its lack in the 6-month-olds. However, if we assume perceptual assimilation, the NRV does not apply to these data since it makes no predictions concerning within-category asymmetries.

To summarize, our results show perceptual reorganization in vowel discrimination between 6 and 9 months of age characterized by the emergence of a perceptual asymmetry at 9 months. In line with assumptions of an early universal discrimination ability, 6-month-olds discriminated the vowels independent of any presentation direction, while the 9-month-olds showed a perceptual asymmetry. The direction of this asymmetry is compatible with both models of asymmetric vowel perception and as already shown for adults (Liu et al., 2021), both phonetic properties of the vowel as well as language-specific features may contribute to perceptual asymmetry and its development during infancy. Much of the previous research in the field of infant vowel perception and its development involves comparisons of the ability to discriminate native versus non-native contrasts. However, we want to point out that in light of the PAM (Tyler et al., 2014), the classification of a vowel contrast as native or non-native is not trivial. Certainly, a native vowel contrast is one that falls into two distinct categories of the native language. However, non-native contrasts can be in different relations to the native language: they can be assimilated to two different categories of the native language and in this case show similar perceptual characteristics as for native contrasts. Alternatively, they can be assimilated to a single native category with or without different degrees of fitting this category. In this case, effects of the internal structure of the native vowel category may affect the perception of the contrast. Future research on infant vowel perception must therefore be more specific in the choice of the vowel contrasts used in the studies.

While our study has provided valuable insights into the emergence of perceptual asymmetries in the perceptual attunement to the native language, it is essential to acknowledge that—beyond the limitations already mentioned in the previous sections—the absence of a native language infant group constitutes a noteworthy gap. The inclusion of Polish-learning infants is essential to further disentangle the potential effects of phonetic properties and language-specific effects on the presence and potential developmental change of asymmetries in vowel perception. Designing studies that can effectively tease apart these multifaceted interactions presents inherent challenges. Our study, while striving to address this issue, could not yet fully capture the entirety of these intricate dynamics which will need future research. However, what our study clearly shows is that potential asymmetries and native language influences have to be considered in depth in any study on the perception of vowels in early language development.

## Notes

1. The calculation of Cohen’s *d* was based on the following formula:

$$\frac{mean\log\left(LT\right)familiar-mean\log\left(LT\right)novel}{\sqrt{{\left(SD\log\left(LT\right)familiar\right)}^{2}-{\left(SD\log\left(LT\right)novel\right)}^{2}}}$$
